# KDM5A controls bone morphogenic protein 2-induced osteogenic differentiation of bone mesenchymal stem cells during osteoporosis

**DOI:** 10.1038/cddis.2016.238

**Published:** 2016-08-11

**Authors:** Chuandong Wang, Jing Wang, Jiao Li, Guoli Hu, Shengzhou Shan, Qingfeng Li, Xiaoling Zhang

**Affiliations:** 1 The Key Laboratory of Stem Cell Biology, Institute of Health Sciences, Shanghai Jiao Tong University School of Medicine (SJTUSM) and Shanghai Institutes for Biological Sciences (SIBS), Chinese Academy of Sciences (CAS), Shanghai, 200025, China; 2 Department of Plastic and Reconstructive Surgery, Shanghai 9th People's Hospital, Shanghai Jiao Tong University School of Medicine, Shanghai, 200011, China; 3 Department of Cell Biology, Zunyi Medical College, Zunyi, 563000, China

## Abstract

Bone morphogenetic protein 2 (BMP2) has been used to induce bone regeneration by promoting osteogenic differentiation of bone marrow-derived mesenchymal stem cells (MSCs). However, its effect is attenuated in osteoporotic conditions by unknown mechanisms. In this study, we investigated the molecular mechanisms of reduced osteogenic effect of BMP2 in osteoporotic conditions. By interrogating the microarray data from osteoporosis patients, we revealed an upregulation of the epigenetic modifying protein lysine (K)-specific demethylase 5A (KDM5A) and decreased Runt-related transcription factor 2 (RUNX2) expression. Further studies were focused on the role of KDM5A in osteoporosis. We first established ovariectomized (OVX) mouse model and found that the BMP2-induced osteogenic differentiation of osteoporotic MSCs was impaired. The elevated level of KDM5A was confirmed in osteoporotic MSCs. Overexpression of KDM5A in normal MSCs inhibited BMP2-induced osteogenesis. Moreover, osteogenic differentiation of osteoporotic MSCs was restored by specific KDM5A short hairpin RNA or inhibitor. Furthermore, by chromatin immunoprecipitation assay we demonstrated that KDM5A functions as endogenous modulator of osteogenic differentiation by decreasing H3K4me3 levels on promoters of Runx2, depend on its histone methylation activity. More importantly, we found an inhibitory role of KDM5A in regulating bone formation in osteoporotic mice, and pretreatment with KDM5A inhibitor partly rescued the bone loss during osteoporosis. Our results show, for the first time, that KDM5A-mediated H3K4me3 modification participated in the etiology of osteoporosis and may provide new strategies to improve the clinical efficacy of BMP2 in osteoporotic conditions.

Osteoporosis, characterized by low bone mineral density and destruction of bone structure, is one of the most common and debilitating skeletal disorders, especially in aged population.^1, 2^ Bone marrow-derived mesenchymal stem cells (MSCs) are common progenitors of osteoblasts and adipocytes in bone. It has been convincingly demonstrated that impaired differentiation of MSCs results in the imbalance between osteogenesis and adipogenesis.^3^ In osteoporosis, adipogenesis in bone marrow is pathologically exuberant but bone formation is significantly decreased.^4^ Restoring the osteogenic differentiation of MSCs is an appealing therapeutic strategy for osteoporosis. Bone morphogenetic protein 2 (BMP2) has been used to induce bone regeneration by promoting osteogenic differentiation of MSCs.^5, 6^ However, extended clinical use of BMP has revealed its transient and low osteo-inductive efficacy *in vivo.*^7, 8^ The molecular mechanism of abnormal endogenous MSCs fate determination remains elusive, which hinders the progress of osteoporosis treatment.

Epigenetic regulation has been found to be a key modulator of stem cells differentiation.^9, 10^ Epigenetic modification, which controls gene expression, could respond to the environmental stimulation (biochemical^11^ or biophysical^12^) to guide cell lineage commitment. Histone acetylation and trimethylated histone 3 lysine 4, 9 and 27 (H3K4me3, H3K9me3 and H3K27me3), are the dominant epigenetic histone signatures.^13^ H3K4me3 is implicated in transcriptional activation, whereas H3K9me3 and H3K27me3 are associated with transcriptional repression.^14^ Histone methylation has been demonstrated to be eliminated by histone demethylases (Kdms).^13, 15^ Recently, H3K27me3 was reported to be a negative conductor of Wnt signaling. H3K27me3 enrichment in promoter regions of Wnt ligands represses Wnt/*β*-catenin signaling, and therefore osteogenic differentiation.^16^ EZH2 and KDM6A, specific Kdm for H3K27me3, were identified to act as an epigenetic switch to regulate MSC lineage specification.^17^ Besides, the expression of histone deacetylase (HDAC) enzymatic activity is demonstrated to be decreased, and the recruitment of HDAC1 to the promoters of osteoblastic marker genes is downregulated during osteogenesis.^18^ Suppression of HDAC activity with HDAC inhibitors accelerates osteogenesis by inducing osteoblastic marker genes, including osteopontin and alkaline phosphatase (ALP).^19^ The critical role of H3K4me3 and its histone methyltransferase Mll3 have also been found in PPAR*γ*-dependent adipogenesis.^20^ But, the role of specific Kdms for H3K4me3, Kdm5a, in regulating MSCs lineage commitment has not been investigated yet.

In this study, we wonder whether lysine (K)-specific demethylase 5A (KDM5A) functions as endogenous modulator of BMP2-induced osteogenic differentiation of MSCs in osteoporosis. We revealed an upregulation of the KDM5A in both osteoporotic patients and animal models, inhibiting MSCs lineage commitment into osteoblasts. Knockdown of Kdm5a restored osteoporotic BMSC cell lineage commitment by increasing H3K4me3 levels on promoter region of Runt-related transcription factor 2 (Runx2), therefore recovered osteogenic differentiation of osteoporotic MSCs both *in vitro* and *in vivo*. We demonstrated that Kdm5a-mediated H3K4me3 modification participates in the etiology of osteoporosis, which may provide new strategies to improve the clinical efficacy of BMP2 and to enhance bone formation under osteoporotic conditions.

## Results

### Osteogenic differentiation and KDMs are regulated in MSCs of osteoporotic patients and ovariectomized (OVX) mice

**Figure 1:**
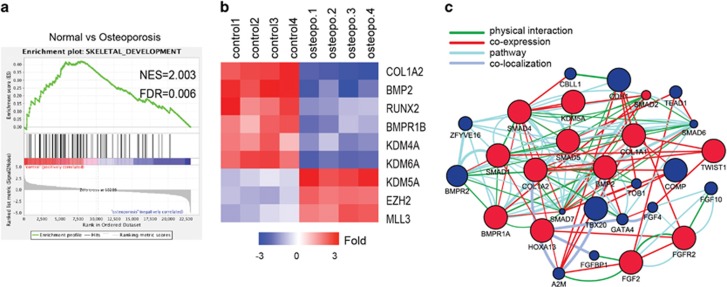
KDM5A expression was increased in MSCs of osteoporosis patients. (**a**) GSEA of expression profiles of MSCs from normal (control) and osteoporosis patients. Enrichment curves computed by GSEA are shown in green (FDR-corrected *P*<0.05). GSEA for skeletal development gene sets demonstrated significant enrichment in control human MSCs as compared with MSCs from osteoporosis patients. (**b**) The heat map is ordered by degree of differential expression of histone methytransferases and demethylases and skeletal development genes between MSCs from normal (control) and osteoporosis patients. (**c**) Identification of molecular functional networks and canonical pathways connected to osteoporosis. The changed genes were analyzed by GeneMANIA with GO-based weighting (i.e., on the basis of molecular function). The BMP2-SMADs pathway is shown as a predominant pathway through the function of its core targets, Red nodes: query genes; blue nodes: highly weighted genes connected to the query genes. Functional networks shown: physical interactions, coexpression, colocalization and pathway networks

It is reported that osteoblastic differentiation of MSCs from osteoporotic patients were impaired by unknown reasons. To investigate underlying mechanisms, we first examined the transcriptomes of MSCs, from four osteoporotic patients and four age-matched normal people (the microarrays were downloaded from the GEO database, GEO accession number GSE35958) using the GSEA method. Consistent with previous reports, we found that skeletal development gene sets were significantly enriched in the MSCs from normal people, but not in those from osteoporotic patients (the NES scores and FDR values were 2.003, 0.06, respectively) (Figure 1a). A heat map for gene products that were differentially expressed by at least twofold shows that osteogenic inducers, such as RUNX2, COL1a2, BMP2 and BMPR1B, were decreased in the osteoporotic group, whereas the expression level of histone demethylase KDM5A for H3K4me3 were increased (Figure 1b). Further dissection through cytoscape and GeneMANIA with GO-based weighting revealed a functional interactive network among the upregulated KDM5A and downregulated SMADs in terms of pathways, physical interactions, coexpression and colocalization parameters (Figure 1c). These results indicate that KDM5A may be involved in impaired osteogensis of osteoporotic MSCs

**Figure 2:**
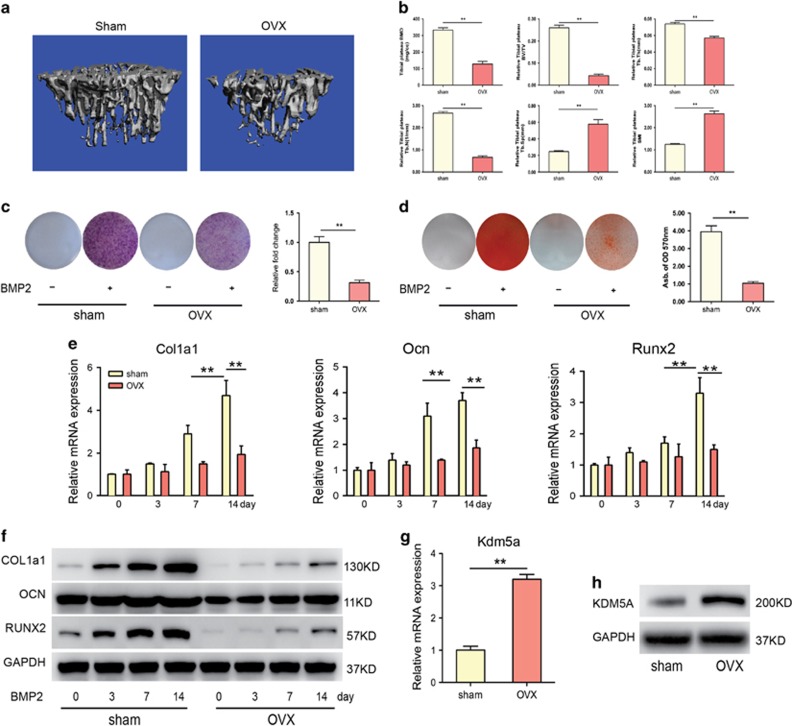
Osteogenic differentiation were decreased and KDM5A expression levels were increased in MSCs of OVX mice. (**a**) Representative *μ*CT reconstructive images of tibial plateau of sham and OVX mice. (**b**) There-dimensional microstructural parameters of tibial plateau of sham and OVX mice. (**c**) ALP activity of MSCs isolated from sham and OVX mice after 7 days of osteogenic induction were detected with ALP staining and quantified. (**d**) Mineralized nodules formed by MSCs isolated from sham and OVX mice after 14 days of osteogenic induction were detected with Alizarin red staining and quantified. (**e**) qRT-PCR analysis of osteogenic differentiation markers Col1a1, Ocn and Runx2 in MSCs isolated from sham and OVX mice after 0, 3, 7 and 14 days osteogenic induction. (**f**) Western blot analysis of Col1a1, Ocn and Runx2 protein accumulation in MSCs isolated from sham and OVX mice after 0, 3, 7 and 14 days osteogenic induction. (**g**) qRT-PCR analysis and (**h**) western blot analysis of Kdm5a in MSCs isolated from sham and OVX mice. All the data were confirmed by three repeated tests. Data were mean±S.D. **P<0.01. All *P-*values are based on Student's *t*-test

Next, we used OVX-induced osteoporotic mouse model to test the role of KDM5A during osteoporosis. A significant decrease in bone mineral density (BMD), trabecular bone volume per tissue volume (BV/TV), trabecular number (Tb.N) and trabecular thickness (Tb.Th) and an increase in trabecular separation (Tb.Sp) and structure model index (SMI) in OVX groups were observed by *μ*CT, confirmed the osteoporotic phenotype of OVX mice (Figures 2a and b). MSCs from sham and OVX mice were isolated to study their osteogenic differentiation ability induced by BMP2. The BMP2-enhanced ALP staining (Figure 2c) and *in vitro* mineralization in MSCs (sham) (Figure 2d), was much more obvious than in MSCs (OVX). Consistent with the above changes, quantitative real-time PCR (qRT-PCR) analysis showed that the expression of the osteogenic marker genes collagen type I alpha 1 (Col1a1), osteocalcin (Ocn) and Runx2 was significantly increased by BMP2 induction in the MSCs (sham) group, whereas the responsiveness of these markers to BMP2 was diminished in MSCs (OVX) (Figure 2e), which was confirmed by the results of the western blot analysis (Figure 2f). However, Kdm5a was significantly increased in MSCs isolated from OVX mice compared with that of sham mice, in accordance with the results of microarrays of osteoporotic patients (Figures 2g and h). It reminded us that the compromised osteogenic differentiation of MSCs of osteoporotic patients or OVX mice may be partly attributed to the altered expression of Kdm5a.

### Kdm5a modulates the osteogenic differentiation of MSCs

**Figure 3:**
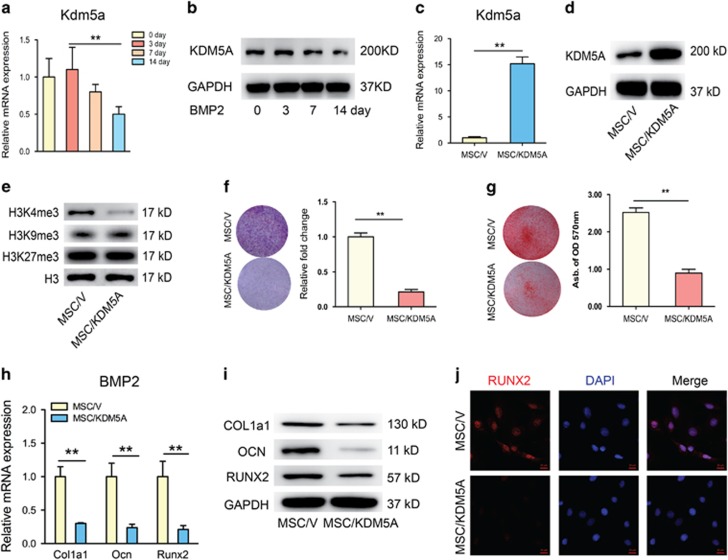
KDM5A overexpression impaired osteogenic differentiation of MSCs. (**a**) qRT-PCR analysis and (**b**) western blot analysis of Kdm5a in MSCs after 0, 3, 7 and 14 days osteogenic induction. (**c**) qRT-PCR analysis and (**d**) western blot analysis of Kdm5a in MSCs after infected with lentiviral vector (MSC/V) and lentiviral-Kdm5a (MSC/KDM5A). (**e**) Western blot analysis of H3K4me3, H3K9me3 and H3K27me3 in MSCs with overexpression of Kdm5a. (**f**) ALP activity of MSCs infected with lentiviral-vector or lentiviral-Kdm5a after 7 days of osteogenic induction were detected with ALP staining and quantified. (**g**) Mineralized nodules formed by MSCs infected with lentiviral-vector or lentiviral-Kdm5a after 14 days of osteogenic induction were detected with Alizarin red staining and quantified. (**h**) qRT-PCR analysis and (**i**) western blot analysis of Col1a1, Ocn and Runx2 expression in MSCs infected with lentiviral-vector or lentiviral-Kdm5a after 7 days of osteogenic induction. (**j**) Immunostaining of RUNX2 (red) location in MSCs infected with lentiviral-vector or lentiviral-Kdm5a after 7 days of osteogenic induction. Scale bar, 20 *μ*m. All the data were confirmed by three repeated tests. Data were mean±S.D. **P<0.01. All *P*-values are based on Student's *t*-test

The expression profile of Kdm5a was found to be decreased during the BMP2-induced osteogenic differentiation (Figures 3a and b). To further investigate the direct role of Kdm5a in osteogenesis of MSCs, we overexpressed it in normal MSCs (Figures 3c and d) and found that KDM5A overexpression decreased the level of H3K4me3 but did not affect H3K9me3, and H3K27me3 (Figure 3e). ALP assays and Alizarin red staining for mineralized deposits showed that Kdm5a overexpression significantly abrogated the BMP2-induced increase in ALP activity and mineralized deposits (Figures 3f and g). Besides, RT-PCR (Figure 3h) and western blot (Figure 3i) examination indicated that KDM5A overexpression also reduced the mRNA and protein levels of Col1a1, Ocn and Runx2 under BMP2 stimulation. Immunofluorescence assay also confirmed that Runx2 expression decreased with KDM5A overexpression (Figure 3j).

**Figure 4:**
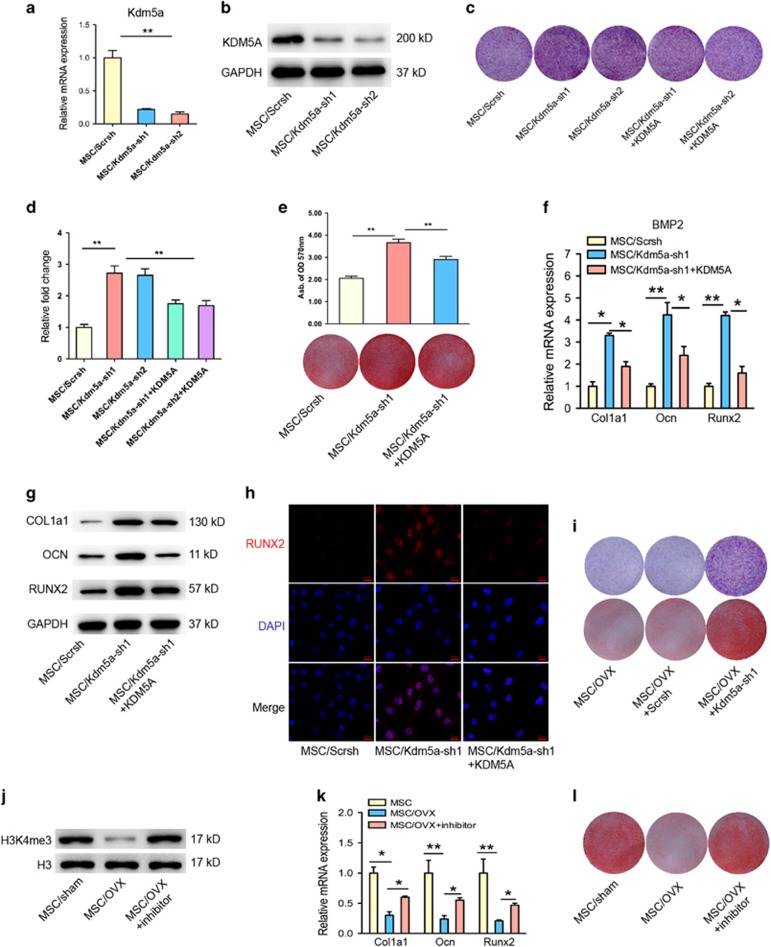
KDM5A knockdown enhanced osteogenic differentiation of MSCs. (**a**) qRT-PCR analysis and (**b**) western blot analysis of Kdm5a in MSCs after infected with lentiviral-Scrsh, lentiviral-Kdm5a-sh1 and lentiviral-Kdm5a-sh2. (**c**) Representative images of ALP staining of MSCs in Scrsh, Kdm5a-sh1, Kdm5a-sh2, Kdm5a-sh1+Kdm5a and Kdm5a-sh2+Kdm5a groups after 7 days of osteogenic induction. (**d**) Quantitative analysis of ALP activity of MSCs in Scrsh, Kdm5a-sh1, Kdm5a-sh2, Kdm5a-sh1+Kdm5a and Kdm5a-sh2+Kdm5a groups after 7 days of osteogenic induction. (**e**) Representative images of Alizarin red staining (including quantitative analysis) of MSCs in Scrsh, Kdm5a-sh1 and Kdm5a-sh1+Kdm5a groups after 14 days of osteogenic induction. (**f**) qRT-PCR analysis and (**g**) western blot analysis of Col1a1, Ocn and Runx2 expression in MSCs in Scrsh, Kdm5a-sh1 and Kdm5a-sh1+Kdm5a groups after 7 days of osteogenic induction. (**h**) Immunostaining of Runx2 (red) location in MSCs in Scrsh, Kdm5a-sh1 and Kdm5a-sh1+Kdm5a groups after 7 days of osteogenic induction. Scale bar, 20 *μ*m. (**i**) Representative images of Alizarin red staining of MSCs isolated from OVX mice in Scrsh, Kdm5a-sh1 groups after 14 days of osteogenic induction. (**j**) Western blot analysis of H3K4me3 expression in MSCs of sham mice and OVX mice with or without Kdm5a inhibitor (JIB-04 with 300 nM) treatment. (**k**) qRT-PCR analysis of the expression of Col1a1, Ocn and Runx2 in MSCs of sham mice and OVX mice with or without Kdm5a inhibitor treatment. (**l**) Representative images of Alizarin red staining of MSCs isolated from sham mice and OVX mice with or without Kdm5a inhibitor treatment. All the data were confirmed by three repeated tests. Data were mean±S.D. *P<0.05,**P<0.01. All *P*-values based on Student's *t*-test

Conversely, we inhibited Kdm5a using specific short hairpin RNAs (shRNAs) in the BMP2-induced osteogenic differentiation model. To rule out off-target effects of shRNA, two different shRNA sequences (Kdm5a-sh1 and Kdm5a-sh2) were used, both of which resulted in efficient depletion of Kdm5a in MSCs as confirmed by RT-PCR and western blot (Figures 4a and b). We found that the BMP2-induced expression of the osteogenic markers Col1a1, Ocn and Runx2 were enhanced by Kdm5a shRNA treatment, which was further supported by the ALP and *in vitro* mineralization staining results (Figures 4c and g). To confirm the specific effects of Kdm5a in osteogenic differentiation of MSCs, we strategically restored its expression in MSCs that already express Kdm5a shRNA. When we restored the Kdm5a expression in Kdm5a knockdown MSCs, the enhanced expression of osteogenic markers Col1a1, Ocn and Runx2 was abrogated significantly, as evidenced by ALP and *in vitro* mineralization staining, RT-PCR, western blot (Figures 4c and g) and immunofluorescence examination (Figure 4h). Furthermore, knockdown of Kdm5a could rescue the impaired osteogenic differentiation in MSCs of OVX mice, shown by *in vitro* mineralization staining (Figure 4i). Meanwhile, the impaired osteogenic differentiation of MSCs isolated from OVX mice was partly rescued by Kdm5a-specific inhibitor (JIB-04, Figure 4j) as Kdm5a shRNA1, as evidenced by RT-PCR and *in vitro* mineralization staining (Figures 4k and l). These results suggest that KDM5A negatively regulate osteoblast differentiation, and that impaired ostegenesis of osteoporotic MSCs because of, at least in part, elevated KDM5A level.

### KDM5A inhibits osteogeninsis by demethylating H3K4me3 on Runx2 promoter region

**Figure 5:**
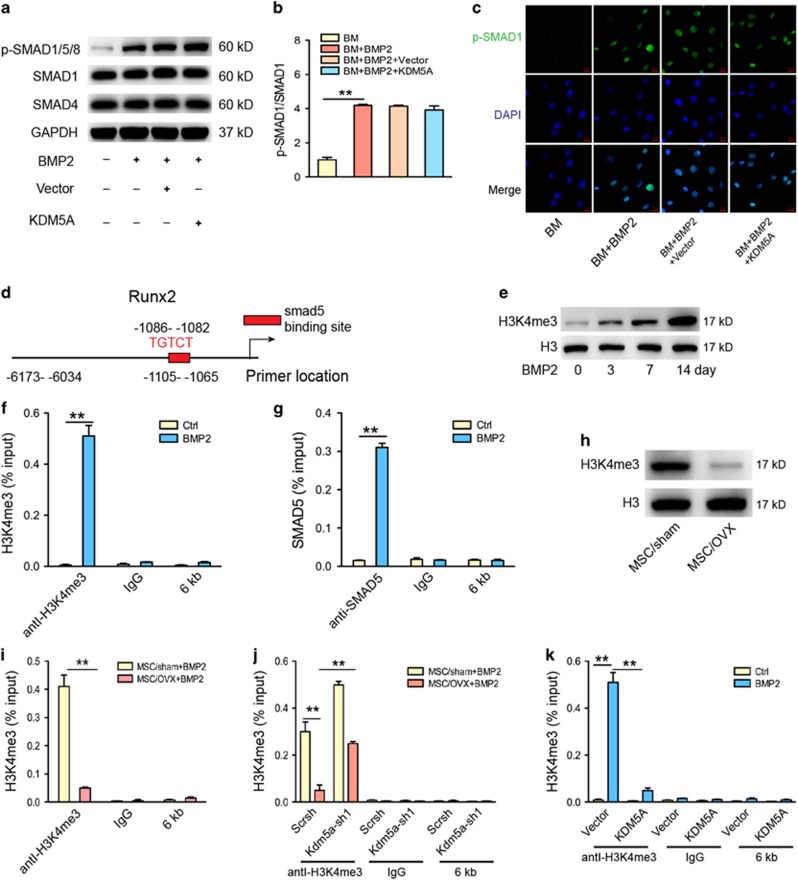
KDM5A inhibited Runx2 expression in MSC by removal of H3K4me3 marks. (**a**) Western blot analysis of p-Smad1/5/8, Smad1 and Smad4 in MSCs infected with lentiviral-vector or lentiviral-Kdm5a after 4 hours of osteogenic induction. (**b**) Quantitative analysis of p-Smad1 expression. Smad1 was used as internal control. (**c**) Immunostaining of p-Smad1/5/8 (green) location in MSCs infected with lentiviral-vector or lentiviral-Kdm5a after 4 hours of osteogenic induction. Scale bar, 20 *μ*m. (**d**) Schematics of Runx2 promoter denoting ChIP-PCR amplified region (−1105 bp to −1065 bp) encompassing the SMAD binding element and the control region 6-kb upstream of the transcription start site (−6173 bp to −6034 bp). (**e**) Western blot analysis of H3K4me3 in MSCs after 0, 3, 7 and 14 days BMP2 treatment. (**f**) Occupancy of H3K4me3 at the Runx2 promoter following BMP2 treatment. (**g**) SMAD5 occupancy at the Runx2 promoter after BMP2 treatment. (**h**) Western blot analysis of H3K4me3 in MSCs of sham and OVX mice. (**i**) Occupancy of H3K4me3 at the Runx2 promoter in MSCs of sham and OVX mice following BMP2 treatment. (**j**) Knockdown of Kdm5a increased the occupancy of H3K4me3 at the Runx2 promoter following BMP2 treatment in MSCs of sham and OVX mice. (**k**) Overexpression Kdm5a decreased the occupancy of H3K4me3 at the Runx2 promoter following BMP2 treatment in MSCs of sham and OVX mice. All the data were confirmed by three repeated tests. Data were mean±S.D. **P<0.01. All *P*-values are based on Student's *t-*test

BMP2 induces osteoblastic differentiation through activating the SMAD1/5/8 signaling pathway.^5, 21^ Thus, we investigated whether KDM5A inhibits BMP2-induced osteoblastic differentiation through altering the BMP2-SMAD1/5/8 pathway in the KDM5A overexpressed MSCs. The results showed that phosphorylated SMAD1/5/8 was obviously increased after BMP2 treatment for 4 h in normal MSCs, which were not significantly changed by KDM5A overexpression (Figure 5a). The total protein level of SMAD1 and SMAD4 all remained unaltered (Figure 5a). The bands intensity were quantified in Figure 5b. Immunofluorescence assay also confirmed that phosphorylated SMAD1/5/8 were translocate into the nucleus after BMP2 induction in normal as well as KDM5A overexpression MSCs (Figure 5c). These results show that Kdm5a modulates the BMP2-induced osteogenic differentiation of MSCs without interrupting the BMP2-SMAD signaling pathway. Kdm5a is a histone demethylase that inhibits the target gene expression levels by removing the epigenetic mark H3K4me3. As we noticed that the expression level of osteogenic key regulator Runx2 was markedly downregulated by KDM5A overexpression, we next examined whether KDM5A regulates MSCs osteogenic differentiation by epigenetically modulating Runx2 expression. Chromatin immunoprecipitation (ChIP) assay was performed to assess the H3K4me3 as well as physical occupancy of KDM5A at the Runx2 promoter region (Figure 5d). After BMP2 treatment, the expression level of H3K4me3 increased (Figure 5e) and we identified a significant increased H3K4me3 level at the Runx2 promoter (Figure 5f). SMAD5 was found to bind to the Runx2 promoter region of MSCs (Figure 5g). However, in BMSC of OVX mice, the expression level of H3K4me3 was markedly decreased (Figure 5h), and the amount of H3K4me3 modification at the Runx2 promoter of MSC/OVX was lower than MSC/sham (Figure 5i). Similarly, in KDM5A overexpressed MSCs, BMP2-induced H3K4me3 level was significantly inhibited (Figure 5j). Most importantly, the impaired H3K4me3 modification in MSCs/OVX could be rescued by Kdm5a inhibitory shRNA1 (Figure 5k). As a control, the level of H3K4me3 remained unchanged at the 6-kb upstream of the transcription start site upon BMP2 treatment in normal MSCs or Kdm5a-modulated MSCs. These findings indicate that H3K4me3 is required for RUNX2 expression in osteogenic differentiation of MSCs, which is inhibited in osteoporotic MSCs by upregulated KDM5A.

### Inhibition of Kdm5a rescued promotes bone formation of OVX mice *in vivo*

**Figure 6:**
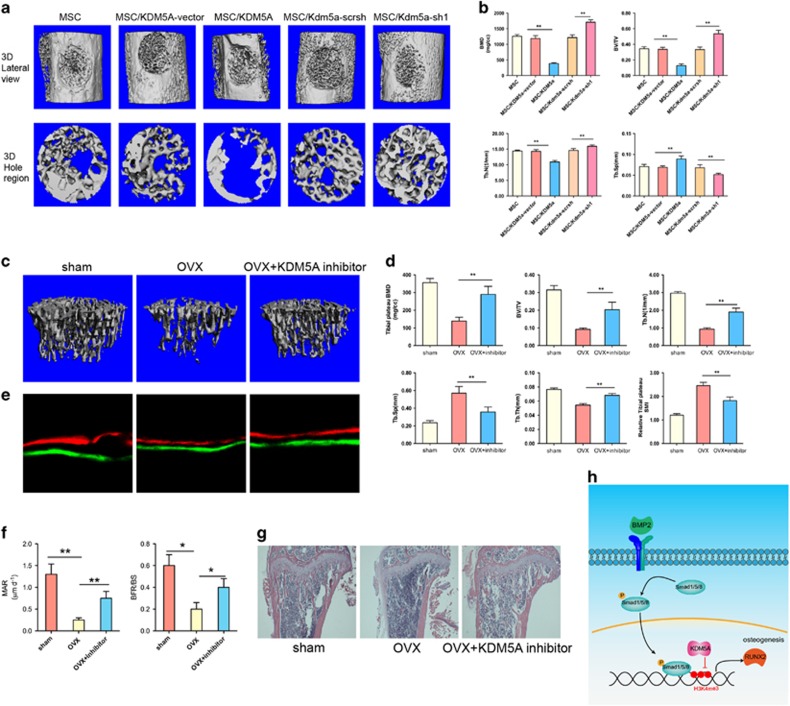
Inhibition of KDM5A rescued the decrease of bone formation *in vivo.* (**a**) Lateral views of 3D reconstruction of injured tibiae (top panel) and mineralized bone formed in hole region (lower panel) by *μ*CT. Representative images from of each group. (**b**) 3D structural parameters-BMD, BV/TV, Tb.N and Tb.Sp-of mineralized bone formed in hole region by *μ*CT. (**c**) Representative images of *μ*CT reconstructive images of tibial plateau in sham, OVX and OVX+Kdm5a inhibitor groups from each group. (**d**) 3D structural parameters-BMD, BV/TV, Tb.N, Tb.Sp, Tb.Th and SMI-of tibial plateau by *μ*CT in sham, OVX and OVX+Kdm5a inhibitor groups. (**e**) Representative images showing new bone formation assessed by Alizarin red and calcein labeling in each group. (**f**) Histomorphometric analysis of MAR and BFR in sham, OVX and OVX+Kdm5a inhibitor groups. (**g**) Representative hematoxylin-eosin staining images of tibial plateau showing bone volume in each group. (**h**) Schematic diagram of the role of Kdm5a in regulating MSCs differentiation and bone formation under mechanical stimulation. All the data were confirmed by three repeated tests. Data were mean±S.D. *P<0.05, ** *P*<0.01. All *P-*values are based on Student's *t*-test

To investigate the function of Kdm5a *in vivo*, we used the tibial monocortical defect model. *μ*CT showed that the ability of bone formation was obviously decreased in KDM5A overexpressed MSCs and increased in MSCs with Kdm5a shRNA1 (Figure 6a), which was confirmed by the results of bone volume-related analysis (Figure 6b). Besides, OVX-induced bone loss was partly rescued by KDM5A inhibitor (JIB-04) (Figure 6c). Bone volume-related parameters such as BMD and BV were also partly increased by KDM5A inhibition (Figure 6d). Similarly, the assessment of bone formation indicated by green fluorescent calcein and red fluorescent Alizarin red and bone volume-related analysis (MAR and BFR) showed that the OVX-induced decrease in bone formation was also rescued by KDM5A inhibition (Figures 6e and f). Furthermore, a histological assessment demonstrated that compared with staining in the sham group, the bone staining of the OVX group was significantly decreased. The overall area of stained bone was increased in the OVX group treated with KDM5A inhibitor, which indicates that the impaired development of new bone was partly rescued by KDM5A inhibition (Figure 6g). Taken together, these results indicate that BMP2 therapeutic inhibition of KDM5A (by shRNA or inhibitor of its methytranferase activity) was able to partly counteract the bone loss observed in osteoporosis (Figure 6h).

## Discussion

The predominant pathway of promoting osteogenesis involves BMP2-SMAD signaling, which leads to the activation of osteoblast essential genes, including Runx2. Previous studies have shown that Runx2 is highly expressed in osteoblasts and osteosarcoma cells. To the contrast, in our study the expression level of Runx2 decreased in MSCs of osteoporotic patients and mice (Figures 1b and 2e). Besides, the BMP2-induced Runx2 expression and osteogenic differentiation was significantly impaired in osteoporotic MSCs than normal (Figures 2e and f). Thus, the mechanism for regulating the expression of Runx2 was unclear, and the discrepancy in BMP2 inducing Runx2 expression between normal and osteoporotic MSCs remain largely unknown. In this study, we compared the transcriptomes of MSCs from osteoporotic patients, and we found that the osteogenic differentiation markers of MSCs, was decreased, whereas KDM5A and EZH2 were increased, which indicated that osteogenic differentiation of MSCs may be controlled by the statues of histone methylation of Runx2.

Gene expression can be regulated at the epigenetic level that includes different modifications of chromatin. Recent studies have indicated that MSC differentiation is sensitive to epigenetic changes.^22^ The application of epigenetic regulators, such as inhibitors of histone modification enzymes, may be valuable for stem cell-based interventions.^23, 24^ Previous studies showed that the degree of H3K9 acetylation and H3K4 trimethylation in MSCs increased, whereas the level of H3K9 trimethylation decreased during osteogenic differentiation,^25^ which was in consistent with our results. For example, TSA, an inhibitor of HDAC1, promoted osteogenic differentiation by altering the epigenetic modifications on the Runx2 promoter in a BMP signaling-dependent manner.^26^ In our study, we examined the roles of Kdm5a in osteogenic differentiation. We performed the gene-silencing experiments by shRNA. Knockdown of Kdm5a increased the expression levels of Runx2, a key regulator of osteoblastic differentiation. Overexpression of Kdm5a decreased ALP activity, bone mineralization, and the expression of Runx2 and Ocn. To further clarify the role of Kdm5a in bone formation *in vivo*, we used tibial monocortical defect model and found that Kdm5a overexpression decreased bone formation of MSCs. Taken together, this information indicates that Kdm5a impaired osteogenic differentiation and bone formation.

Recently, the role of Kmts and Kdms in regulating MSC lineage specification has been widely reported.^15, 27^ Kdm4b, specifically demethylates Lys-9 of histone H3, was upregulated by bone morphogenetic protein. This BMP-induced Kdm4b has a critical role in MSC osteogenic differentiation by removing H3K9me3 silencing marks at the Dlx5 promoter.^28^ Kdm4b was also required for recruitment of SMAD3 and Sox9 activation during TGF-*β*-mediated chondrogenic differentiation of MSCs.^29^ Interestingly, G9a, H3K9 methyltransferase, was found to repress adipogenesis by inhibiting PPAR*γ* expression and facilitating Wnt10a expression independent of its enzymatic activity.^30^ In a prostate cancer cell line, it was found that Runx2 target genes required recruitment of G9a for their expression, but did not depend on its histone methyltransferase activity.^25^ These studies indicates that Kmts G9a may function as a coregulator of other transcription factors to control lineage specification of MSCs independent of its enzymatic activity, but not as the Kdm4b. But, it was still obscure that whether G9a would interplay with SMADs to regulate the Runx2 expression. Another repressive histone modification H3K27me3 was demonstrated to regulate osteogenic differentiation and Runx2 expression of MSCs.^27^ Enhancer of Zeste homology 2 (EZH2), the catalytic component of polycomb repressive complex 2, catalyzes the trimethylation on histone 3 lysine 27 (H3K27me3).^16^ Previous study demonstrated that redundant EZH2 shifted MSC differentiaton to adipocyte, which contributed to the development of osteoporosis by increasing the level of H3K27me3 on the promoter regions of Wnt1, Wnt6 and Wnt10a.^16, 17^ Similarly, Kdm6a, which specifically catalyzes the removal of trimethylation of H3K27me3, was induced by the stimulation of osteogenic differentiation as well as treatment of BMP2. Silencing of Kdm6a decreased the promoter activities of Runx2 by increasing the level of H3K27me3 on the promoter regions and therefore suppressed osteogenic differentiation.^31^ Although Kdms of repressive histone modifications H3K9me3 (Kdm4b) and H3K27me3 (Kdm6a) were demonstrated to promote osteogenic differentiation. In this study, Kdm for promotative histone modification H3K4me3 (Kdm5a) was found to inhibit osteogenic differentiation of MSCs by removing H3K4me3 activating marks at the Runx2 promoter. Kdm5a was upregulated in osteoporotic MSCs, and Kdm5a overexpression blocked BMP2-induced osteogenic differentiation. Inhibitor of the enzymatic activity of Kdm5a (JIB-04) rescued the impaired bone formation in OVX mice, which further confirmed that Kdm5a controls osteogenic differentiation and Runx2 expression dependent on its histone methyltransferase activity to reduce H3K4me3 mark.

Our study also showed that SMAD5 binding onto the Runx2 promoter upon BMP2 treatment was repressed by Kdm5a, as Kdm5a overexpression resulted in decreased occupancy of SMAD5 at the Runx2 promoter (data not shown). Therefore, the physical presence of SMAD5 in the nucleus is not sufficient to activate Runx2 expression, but additional regulatory mechanisms involving Kdm5a are required. Removing H3K4me3 on the Runx2 promoter by Kdm5a may inhibit the recruitment of SMAD5 and other transcriptional coactivators to the Runx2 promoter. Besides, by GeneMANIA with GO-based weighting, we revealed a functional interactive network among the Kdm5a and SMADs (Figure 1c). It has been demonstrated that Mll3 and Mll4, Kmts of H3K4me3, were recruited to the PPAR*γ-*activated aP2 gene during adipogenesis, and was shown to interact directly with PPAR*γ*.^20^ However, in this study, we did not investigate whether Mll3 was involved in SMAD5 activated Runx2 expression during osteogenesis, which needs in-depth researches. Interestingly, in this study, we found that although Kdm5a was downregulated during osteogenic differentiation (Figures 3a and b), BMP2 did not change the expression of Kdm5a during 72 h. Previous study showed that TGF-*β* decreased KDM4B expression and therefore promoted chondrogenic differentiation.^29^ So, it demands more meticulous researches to identify the original causes to explain the Kdm5a expression pattern in osteoporosis.

## Materials and Methods

### Microarray analysis and gene set enrichment analysis (GSEA)

The microarrays for human MSCs of elderly individuals or patients with osteoporosis (GSE35959) were obtained from the GEO database. Normalization of the expression profiles was performed by dividing values by the mean signal of each array representing a single sample using GeneSight-Lite 4.1.6 (BioDiscovery, El Segundo, CA, USA). C2 curated gene sets and C5 GO gene sets, downloaded from Molecular Signatures Database (MSigDB by Broad Institute, Cambridge, MA, USA), were tested for enrichment based on the GSEA method.^32^ Genes were sorted according to the value of the t-statistic computed against the human BMSC 'Normal (control) *versus* Osteoporosis (test)' phenotype, with genes upregulated in the 'control' class at the left-end of the list and genes upregulated in the 'test' class at the right-end of the list. Skeletal development genes were located within the sorted list. The resulting heat map and the intensity data were partly inspected for genes differentially expressed between the MSCs of normal donors and those with osteoporosis. The molecular functional networks and canonical pathways of the genes upregulated in osteoporosis were analyzed by the GeneMANIA Cytoscape plugin.^33^ GO-based weighting (molecular function based) was used for calculations. The functional networks, including coexpression, physical interaction, pathway and predicted networks, were analyzed.

### OVX animal model

The procedures used in this study were as humane as possible. The animal study protocol was reviewed and approved by the institution's Animal Ethics Committee of the Shanghai Jiao Tong University School of Medicine (SJTUSM). Twenty-four healthy 8-week-old C57BL/J6 female mice were assigned to three groups of eight mice each randomly: control (sham), surgical ovariectomy (OVX) and OVX+Kdm5a inhibitor groups. The mice were allowed to acclimate to their environment for 1 week before surgery. Then, the mice were either dorsal OVX or sham operated (sham) under anesthetization with chloral hydrate. Starting from 3-week post-surgery, OVX+Kdm5a inhibitor group were treated with JIB-04 (55 mg/kg), whereas the sham and OVX groups were treated with vehicle by oral administration. Four weeks after drug administration, tibial plateau were harvested and the structure were measured with a SCANCO Medical *μ*CT 40 scanner. The images were analyzed using SCANCO evaluation software to perform segmentation, conduct a three-dimensional morphometric analysis, and determine the density and distance parameters (SCANCO Medical AG, Zurich, Switzerland). The three-dimensional structural parameters analyzed included the following: TV (total tissue volume, containing both trabecular and cortical bone), BV/TV, Tb.Th, Tb.Sp and SMI. For the assessment of new bone formation, we injected green fluorescent calcein (Sigma; 5 mg/kg body weight) and red fluorescent Alizarin red (Sigma, St. Louis, MO, USA,10 mg/kg) into the mice on days 7 and 2 before killing. Bone histomorphometric analyses mineral apposition rate (MAR) and bone formation rate (BFR/BS) were performed using professional image analysis software (Image J, NIH, Bethesda, MD, USA) under fluorescence microscopy (Leica, Q500MC, Solms, Germany). The bone histomorphometric parameters were calculated and expressed according to the standardized nomenclature for bone histomorphometry.

### Cell culture

Bone MSCs from the femurs of mice were flushed out with *α*-minimum essential medium (*α*-MEM, Hyclone by Thermo Fisher Scientific, Waltham, MA, USA) and cultured in growth medium (*α*-MEM including 10% fetal bovine serum (Gibco by GE Healthcare Life Sciences, Logan, UT, USA) and 1% penicillin and streptomycin (Hyclone)) at 37 °C in the presence of 5% CO_2_. Non-adherent cells were removed by replacing the medium after 3 days. The attached MSCs were used for experiments at passages 3 to 7.

### Lentiviral transduction overexpression studies

Kdm5a gene was ligated into pLVX-IRES-purousing to construct KDM5A overexpression plasmid. The pLVX-IRES-puro and pRUF-IRES-puro-Kdm5a constructs were transfected into the HEK293T viral packaging cell line together with the psPAX2 and pMD2.G plasmids. Forty-eight hours after transfection, the viral supernatant was collected and used for the infection of MSCs.

### shRNA knockdown studies

The pLKO.1-lentiviral shRNA vectors that target Kdm5a and nonsilencing pLKO.1 control vector (Scrsh) were synthesized by Open Biosystems (Thermo Fisher Scientific, Inc., Waltham, MA, USA). The lentiviruses were packaged via the co-transduction HEK293T cells with the lentiviral vector plasmid containing shRNA and the packaging plasmids psPAX2 and pMD2.G using Lipofectamine 2000 transfection reagent (Invitrogen, Carlsbad, CA, USA). Forty-eight hours after transfection, the viral supernatant was collected and used for the infection of MSCs.

### ALP staining

ALP staining was performed according to the manufacturer's instructions. Briefly, cell layer was rinsed with phosphate-buffered saline (PBS) three times, followed by fixation in 4% paraformaldehyde for 10 min at room temperature. The cells were then incubated with buffer containing 0.1% naphthol AS-Bi phosphate (Sigma-Aldrich, St. Louis, MO, USA) and 2% fast violet B (Sigma-Aldrich). After incubation for 1 h at 37 °C, the cell layer was washed with deionized water.

### ALP activity assay

MSCs were scrapped from the dishes and suspended in double-distilled H_2_O (ddH_2_O) before freezing and thawing for three times. ALP activity was determined at 405 nm using p-nitrophenyl phosphate (pNPP) (Sigma-Aldrich) as the substrate. A 50 ml of sample was mixed with 50 ml of pNPP (1 mg/ml) in 1 m diethanolamine buffer containing 0.5 mM MgCl_2_ (pH 9.8) and incubated at 37 °C for 15 min on a bench shaker. The reaction was stopped by adding of 200 ml of 2 m NaOH per 200 *μ*l of reaction mixture. Total protein content was determined by the BCA method with protein assay kit (Pierce, Rockford, IL, USA). ALP activity was calculated as nmol p-nitrophenol per minute per mg protein, and presented as fold changes over control group.

### Alizarin red staining

Cells were fixed in 70% ice-cold ethanol for 1 h and rinsed with ddH_2_O. Cells were then stained with 40 mM Alizarin red S (pH 4.9, Sigma) for 15 min with gentle agitation. After staining, cells were rinsed five times with ddH_2_O. For the quantitative assessment of the degree of mineralization, the red stain was eluted by 10% (w/v) cetylpyridinium chloride (Sigma-Aldrich) for 1 h and quantified via spectrophotometric absorbance measurements of OD at 570 nm.

### RNA purification and qRT-PCR

The total RNA of cells was isolated using TRIzol reagent (Invitrogen) according to the manufacturer's instructions. After the reverse transcription reaction, real-time PCR was performed with an ABI 7900HT system using SYBR Premix (Takara, Dalian, China) according to the manufacturer's instructions. The conditions of the real-time PCR were as follows: denaturation at 95 °C for 10 s, 40 cycles at 95 °C for 10 s and 60 °C for 30 s. A dissociation stage was added to the end of the amplification procedure. No nonspecific amplification was observed, as determined using the dissociation curve. Glyceraldehyde 3-phosphate dehydrogenase (GAPDH) was used as an internal control. The data were analyzed using the comparison Ct (2^−ΔΔCt^) method and expressed as the fold change relative to the respective control. Each sample was analyzed in triplicate. The primer sequences used in this study were as follows: GAPDH: forward, 5′-AGGTCGGTGTGAACGGATTTG-3′ reverse, 5′-GGGGTCGTTGATGGCAACA-3′ Runx2: forward, 5′-GACTGTGGTTACCGTCATGGC-3′ reverse, 5′-ACTTGGTTTTTCATAACAGCGGA-3′ Col1a1: forward, 5′-GCTCCTCTTAGGGGCCACT-3′ reverse, 5′-ATTGGGGACCCTTAGGCCAT-3′ Ocn: forward, 5′-GACAAGTCCCACACAGCAACT-3′ reverse, 5′-GGACATGAAGGCTTTGTCAGA-3′ Kdm5a: forward, 5′-CACAGACCCGCTGAGTTTTAT-3′ reverse, 5′-CTTCACAGGCAAATGGAGGTT-3′.

### Western blot analysis

For the western blot analysis, cells were lysed on ice for 30 min in a lysis buffer containing 50 mM Tris–HCl (pH 7.4), 150 mM NaCl, 1% Nonidet P-40 and 0.1% SDS supplemented with protease inhibitors (10 mg/ml leupeptin, 10 mg/ml pepstatin A and 10 mg/ml aprotinin). Protein fractions were collected by centrifugation at 15 000 *g* at 4 °C for 10 min and then subjected to 10% SDS-PAGE and transferred to polyvinylidene difluoride membranes. The membranes were blocked with 5% BSA and incubated with specific antibodies overnight at 4 °C. A horseradish peroxidase-labeled secondary antibody was added and visualized using an enhanced chemiluminescence detection system (Millipore, Billerica, MA, USA) as recommended by the manufacturer. We used the following primary antibodies to determine the concentrations of proteins in the lysates: anti-Runx2, anti-Ocn, anti-Col1a1 mAb (1:1000, Abcam, Cambridge, UK), anti-Kdm5a, anti-GAPDH mAb, anti-Histone H3, anti-H3K4me3, anti-H3K9me3, anti-H3K27me3 and anti-smad1/5/8 mAb (1 : 1000, Cell Signaling Technology, Inc., Danvers, MA, USA).

### Immunofluorescence

MSCs cultured in 35mm cell culture dish (NEST Biotech, Wuxi, Jiangsu, China) were fixed with 4% PFA in PBS for 20 min at room temperature. After washing in PBS, samples were permeabilized with 0.5% Triton X-100 for 5 min and blocked with 5% BSA for 60 min. Incubation with primary anti-p-Smad1/5/8 and anti-Runx2 antibodies (Abcam) was performed overnight at 4 °C. The primary antibodies were detected using FITC or PE-conjugated anti-rabbit or mouse IgG secondary antibodies. After the final wash, the nuclei were counterstained by adding a 2  mg/ml solution of 4',6-diamidino-2-phenylindole (Sigma-Aldrich) in PBS for 10 min before imaging. Cells were visualized using a confocal microscope (Leica).

### ChIP assay

ChIP assays were performed using an EZ ChIP Chromatin Immunoprecipitation Kit (Millipore by Merck Life Science,Temecula, CA, USA) according to the manufacturer's instructions. Briefly, cells cultured under the previously indicated conditions were fixed in 1% formaldehyde/PBS for 10 min at room temperature. After two washes with PBS, cells were resuspended in 0.5 ml of lysis buffer containing a protease inhibitor cocktail before sonication. DNA fragments from the soluble chromatin preparations were 400–800 bp in length. Immunoprecipitation (IP) was carried out overnight with purified anti-H3K4me3 and SMAD5 antibody (Millipore, Bedford, MA, USA) or normal mouse IgG as a negative control. Protein A/G agarose was used to pulldown the antigen–antibody compounds and then washed four times with washing buffers. The DNA–protein crosslinks were reversed with 5 m NaCl at 65 °C for 6 h, and DNA from each sample was purified. PCR was performed using 2 *μ*l DNA samples with the following primers: Runx2 primer: forward, 5′-TTCTAGGTGAAGCCAGGTGGAG-3′ reverse, 5′-AATACAAAAATTAGCTGGGCGTG-3′ 6-kb primer: forward, 5′-AATCTCTTGACCTCGTGATCCACC-3′ reverse, 5′-AGCGACAACCTGGGTGTTTCAAT-3′.

### Tibial monocortical defect model

The tibial monocortical defect model were constructed as previously described.^34^ Eight-week-old C57BL/J6 female mice were assigned to five groups of eight mice each randomly: MSC, MSC+KDM5A vector, MSC+KDM5A, MSC+scramble shRNA and MSC+Kdm5a shRNA groups under anesthesia using chloral hydrate. The lateral aspect of right tibia was exposed and carefully cleared of overlying soft tissues while preserving the PO. A monocortical osseous hole (0.8 mm diameter) was created on the anterior surface of the tibia crest using a round burr attached to a dental drill. Irrigation with saline was used to remove bone dust and fragments. MSC (5 × 10^4^ cells per sample) at passage 3 were resuspended in a mixture of medium and Matrigel (BD Bioscience, San Jose, CA, USA) and the transplanted to the osseous hole. After 3 weeks, tibiae were isolated, fixed overnight in 4% paraformaldehyde and new bone formation were analyzed using SCANCO Medical *μ*CT 40 scanner with a spatial resolution of 5 *μ*m. Three-dimensional structural parameters analyzed included the following: TV (total tissue volume, containing both trabecular and cortical bone), BV/TV and Tb.Sp.

### Statistical analysis

The data are presented as the mean±s.d. (*n* is the number of tissue preparations, cells or experimental replicates). For comparing groups of data, a two-tailed Student's *t*-test was used. A value of *P*<0.05 was considered to be statistically significant.

## Conclusion

In this study, we found that histone demethylase KDM5A is highly expressed in osteoporotic MSCs, which inhibits Runx2 expression by demethylating H3K4me3 at its promoter region. Our results explained, at lease in part, the reduced responsiveness of osteoporotic MSCs to BMP2, and suggested a new therapeutic strategy to improve the clinical efficacy of BMP2 for the treatment of osteoporosis.

## Abbreviations

KDM5A: lysine (K)-specific demethylase 5A
BMP2: bone morphogenetic protein 2
MSCs: mesenchymal stem cells
RUNX2: Runt-related transcription factor 2
OVX: ovariectomized
ChIP: chromatin immunoprecipitation
GSEA: gene set enrichment analysis
ALP: alkaline phosphatase
qRT-PCR: quantitative real-time polymerase chain reaction
Col1a1: collagen type I alpha 1
Ocn: osteocalcin
shRNA: short hairpin RNA
PBS: phosphate-buffered saline
BMD: bone mineral density
BV/TV: trabecular bone volume per tissue volume
Tb.Th: trabecular thickness
Tb.N: trabecular number
Tb.Sp: trabecular separation
SMI: structure model index
MAR: mineral apposition rate
BFR: bone formation rate

## References

[bib1] Bergman RJ, Gazit D, Kahn AJ, Gruber H, McDougall S, Hahn TJ. Age-related changes in osteogenic stem cells in mice. J Bone Mineral Res 1996; 11: 568–577. 10.1002/jbmr.5650110504 9157771

[bib2] D'Ippolito G, Schiller PC, Ricordi C, Roos BA, Howard GA. Age-related osteogenic potential of mesenchymal stromal stem cells from human vertebral bone marrow. J Bone Mineral Res 1999; 14: 1115–1122. 10.1359/jbmr.1999.14.7.1115 10404011

[bib3] Bianco P, Robey PG, Simmons PJ. Mesenchymal stem cells: revisiting history, concepts, and assays. Cell Stem Cell 2008; 2: 313–319. 18397751 10.1016/j.stem.2008.03.002 PMC2613570

[bib4] Justesen J, Stenderup K, Ebbesen EN, Mosekilde L, Steiniche T, Kassem M. Adipocyte tissue volume in bone marrow is increased with aging and in patients with osteoporosis. Biogerontology 2001; 2: 165–171. 11708718 10.1023/a:1011513223894

[bib5] Rosen V. BMP2 signaling in bone development and repair. Cytokine Growth Factor Rev 2009; 20: 475–480. 19892583 10.1016/j.cytogfr.2009.10.018

[bib6] McKay WF, Peckham SM, Badura JM. A comprehensive clinical review of recombinant human bone morphogenetic protein-2 (INFUSE Bone Graft). Int Orthop 2007; 31: 729–734. 17639384 10.1007/s00264-007-0418-6 PMC2266665

[bib7] Fu R, Selph S, McDonagh M, Peterson K, Tiwari A, Chou R et al. Effectiveness and harms of recombinant human bone morphogenetic protein-2 in spine fusion: a systematic review and meta-analysis. Ann Intern Med 2013; 158: 890–902. 23778906 10.7326/0003-4819-158-12-201306180-00006

[bib8] Hoffmann MF, Jones CB, Sietsema DL. Recombinant human bone morphogenetic protein-2 (rhBMP-2) in posterolateral lumbar spine fusion: complications in the elderly. J Orthop Surg Res 2013; 8: 1. 23317417 10.1186/1749-799X-8-1 PMC3621610

[bib9] Ezhkova E, Pasolli HA, Parker JS, Stokes N, Su IH, Hannon G et al. Ezh2 orchestrates gene expression for the stepwise differentiation of tissue-specific stem cells. Cell 2009; 136: 1122–1135. 19303854 10.1016/j.cell.2008.12.043 PMC2716120

[bib10] Lian JB, Stein GS, Javed A, van Wijnen AJ, Stein JL, Montecino M et al. Networks and hubs for the transcriptional control of osteoblastogenesis. Rev Endocr Metab Disord 2006; 7: 1–16. 17051438 10.1007/s11154-006-9001-5

[bib11] Li J, Zhang N, Huang X, Xu J, Fernandes JC, Dai K et al. Dexamethasone shifts bone marrow stromal cells from osteoblasts to adipocytes by C/EBPalpha promoter methylation. Cell Death Dis 2013; 4: e832. 24091675 10.1038/cddis.2013.348 PMC3824658

[bib12] Delaine-Smith RM, Reilly GC. Mesenchymal stem cell responses to mechanical stimuli. Muscles Ligaments Tendons J 2012; 2: 169–180. 23738294 PMC3666521

[bib13] Shi Y. Histone lysine demethylases: emerging roles in development, physiology and disease. Nat Rev Genet 2007; 8: 829–833. 17909537 10.1038/nrg2218

[bib14] Rivenbark AG, Strahl BD. Molecular biology. Unlocking cell fate. Science 2007; 318: 403–404. 17947570 10.1126/science.1150321

[bib15] Pedersen MT, Helin K. Histone demethylases in development and disease. Trends Cell Biol 2010; 20: 662–671. 20863703 10.1016/j.tcb.2010.08.011

[bib16] Jing H, Liao L, An Y, Su X, Liu S, Shuai Y et al. Suppression of EZH2 prevents the shift of osteoporotic MSC fate to adipocyte and enhances bone formation during osteoporosis. Mol Ther 2016; 24: 217–229. 26307668 10.1038/mt.2015.152 PMC4817806

[bib17] Hemming S, Cakouros D, Isenmann S, Cooper L, Menicanin D, Zannettino A et al. EZH2 and KDM6A act as an epigenetic switch to regulate mesenchymal stem cell lineage specification. Stem Cells 2014; 32: 802–815. 24123378 10.1002/stem.1573

[bib18] Lee HW, Suh JH, Kim AY, Lee YS, Park SY, Kim JB. Histone deacetylase 1-mediated histone modification regulates osteoblast differentiation. Mol Endocrinol 2006; 20: 2432–2443. 16728531 10.1210/me.2006-0061

[bib19] Maroni P, Brini AT, Arrigoni E, de Girolamo L, Niada S, Matteucci E et al. Chemical and genetic blockade of HDACs enhances osteogenic differentiation of human adipose tissue-derived stem cells by oppositely affecting osteogenic and adipogenic transcription factors. Biochem Biophys Res Commun 2012; 428: 271–277. 23085045 10.1016/j.bbrc.2012.10.044

[bib20] Lee J, Saha PK, Yang QH, Lee S, Park JY, Suh Y et al. Targeted inactivation of MLL3 histone H3-Lys-4 methyltransferase activity in the mouse reveals vital roles for MLL3 in adipogenesis. Proc Natl Acad Sci USA 2008; 105: 19229–19234. 19047629 10.1073/pnas.0810100105 PMC2614744

[bib21] Yang G, Yuan G, Li X, Liu P, Chen Z, Fan M. BMP-2 induction of Dlx3 expression is mediated by p38/Smad5 signaling pathway in osteoblastic MC3T3-E1 cells. J Cell Physiol 2014; 229: 943–954. 24647893 10.1002/jcp.24525

[bib22] Stein GS, van Wijnen AJ, Imbalzano AN, Montecino M, Zaidi SK, Lian JB et al. Architectural genetic and epigenetic control of regulatory networks: compartmentalizing machinery for transcription and chromatin remodeling in nuclear microenvironments. Crit Rev Eukaryot Gene Expr 2010; 20: 149–155. 21133844 10.1615/critreveukargeneexpr.v20.i2.50 PMC3066044

[bib23] Haberland M, Montgomery RL, Olson EN. The many roles of histone deacetylases in development and physiology: implications for disease and therapy. Nat Rev Genet 2009; 10: 32–42. 19065135 10.1038/nrg2485 PMC3215088

[bib24] Paino F, La Noce M, Tirino V, Naddeo P, Desiderio V, Pirozzi G et al. Histone deacetylase inhibition with valproic acid downregulates osteocalcin gene expression in human dental pulp stem cells and osteoblasts: evidence for HDAC2 involvement. Stem Cells 2014; 32: 279–289. 24105979 10.1002/stem.1544 PMC3963447

[bib25] Purcell DJ, Khalid O, Ou CY, Little GH, Frenkel B, Baniwal SK et al. Recruitment of coregulator G9a by Runx2 for selective enhancement or suppression of transcription. J Cell Biochem 2012; 113: 2406–2414. 22389001 10.1002/jcb.24114 PMC3350606

[bib26] Hu X, Zhang X, Dai L, Zhu J, Jia Z, Wang W et al. Histone deacetylase inhibitor trichostatin A promotes the osteogenic differentiation of rat adipose-derived stem cells by altering the epigenetic modifications on Runx2 promoter in a BMP signaling-dependent manner. Stem Cells Dev 2013; 22: 248–255. 22873791 10.1089/scd.2012.0105

[bib27] Agger K, Christensen J, Cloos PA, Helin K. The emerging functions of histone demethylases. Curr Opin Genet Dev 2008; 18: 159–168. 18281209 10.1016/j.gde.2007.12.003

[bib28] Ye L, Fan ZP, Yu B, Chang J, Al Hezaimi K, Zhou XD et al. Histone demethylases KDM4B and KDM6B promote osteogenic differentiation of human MSCs. Cell Stem Cell 2012; 11: 50–61. 22770241 10.1016/j.stem.2012.04.009 PMC3392612

[bib29] Lee HL, Yu B, Deng P, Wang CY, Hong C. Transforming growth factor-beta-induced KDM4B promotes chondrogenic differentiation of human mesenchymal stem cells. Stem Cells 2016; 34: 711–719. 26485430 10.1002/stem.2231 PMC4858413

[bib30] Wang L, Xu S, Lee JE, Baldridge A, Grullon S, Peng W et al. Histone H3K9 methyltransferase G9a represses PPARgamma expression and adipogenesis. EMBO J 2013; 32: 45–59. 23178591 10.1038/emboj.2012.306 PMC3545301

[bib31] Yang D, Okamura H, Nakashima Y, Haneji T. Histone demethylase Jmjd3 regulates osteoblast differentiation via transcription factors Runx2 and osterix. J Biol Chem 2013; 288: 33530–33541. 24106268 10.1074/jbc.M113.497040 PMC3837102

[bib32] Subramanian A, Tamayo P, Mootha VK, Mukherjee S, Ebert BL, Gillette MA et al. Gene set enrichment analysis: a knowledge-based approach for interpreting genome-wide expression profiles. Proc Natl Acad Sci USA 2005; 102: 15545–15550. 16199517 10.1073/pnas.0506580102 PMC1239896

[bib33] Mostafavi S, Ray D, Warde-Farley D, Grouios C, Morris Q. GeneMANIA: a real-time multiple association network integration algorithm for predicting gene function. Genome Biol 2008; 9(Suppl 1): S4. 10.1186/gb-2008-9-s1-s4 PMC2447538 18613948

[bib34] Hu K, Olsen BR. Osteoblast-derived VEGF regulates osteoblast differentiation and bone formation during bone repair. J Clin Invest 2016; 126: 509–526. 26731472 10.1172/JCI82585 PMC4731163

